# Supplementary material for: Machine learning prediction of future amyloid beta positivity in amyloid-negative individuals
Source: Alzheimers Res Ther. 2024 Feb 27;16:46. doi: 10.1186/s13195-024-01415-w (PMC10900722; doi:10.1186/s13195-024-01415-w)
Supplement: Supplementary file 2 — Supplementary material 2. [file 13195_2024_1415_MOESM2_ESM.pdf]

Supplement to Machine learning prediction of future amyloid beta positivity in amyloid-negative individuals

Elaheh Moradi, Mithilesh Prakash, Anette Hall, Alina Solomon, Bryan Strange, Jussi Tohka

February 26, 2024

Supplementary Tables

Table S1: Summarizing the experiments reported in different subsections in Section 3

Subsection	Prediction Target	Predictors
Predicting PET and CSF Aβ-positivity in Aβ-negative individuals from multimodal data excluding PET and CSF baseline measures	CSF-based Aβ-positivity	Demographics, APOE4, baseline neuropsychological test results and baseline MRI biomarkers
Predicting PET and CSF Aβ-positivity in Aβ-negative individuals from multimodal data excluding PET and CSF baseline measures	PET-based Aβ-positivity	Demographics, APOE4, baseline neuropsychological test results and baseline MRI biomarkers
Predicting PET and CSF Aβ-positivity in Aβ-negative individuals from multimodal data including PET and CSF baseline measures	CSF-based Aβ-positivity	Demographics, APOE4, baseline neuropsychological test results and baseline MRI biomarkers, baseline CSF measures
Predicting PET and CSF Aβ-positivity in Aβ-negative individuals from multimodal data including PET and CSF baseline measures	PET-based Aβ-positivity	Demographics, APOE4, baseline neuropsychological test results and baseline MRI biomarkers, baseline PET measures
Predicting CSF-based future Aβ-positivity from CSF and PET baseline measures	CSF-based Aβ-positivity	Baseline CSF and baseline PET measures
Predicting PET-based future Aβ-positivity from CSF and PET baseline measures	PET-based Aβ-positivity	Baseline CSF and baseline PET measures
MCI/dementia conversion prediction	Future MCI/dementia	Demographics, APOE4, baseline neuropsychological test results and baseline MRI biomarkers

Table S2: The correlation between cognitive measures to the labels in CSF-cohort and PET-cohort (Aβ-Stable vs. Aβ-Converter, and to the labels in regression cohort of CSF (Aβ42) and PET(global SUVR).

	CDRSB	ADAS13	ADASQ4	MMSE	RAVLT-immediate	RAVLT-learning	RAVLT-forgetting	RAVLT_perc-forgetting	LDELTOTAL	TRABSCORE	FAQ
**CSF-cohort**	0.24	0.18	0.15	-0.22	-0.19	-0.12	0.11	0.20	-0.21	0.21	0.20
**PET-cohort**	-0.1	-0.06	-0.08	-0.13	-0.03	-0.01	0.03	0.002	0.04	0.06	-0.06
**CSF, Regression-cohort**	-0.26	-0.30	-0.29	0.19	0.23	0.13	-0.17	-0.27	0.29	-0.18	-0.22
**PET, Regression-cohort**	0.22	0.18	0.16	-0.13	-0.18	-0.06	0.13	0.18	-0.23	0.22	0.22

Table S3: The correlation between MRI measures (volumes) to the labels in CSF-cohort and PET-cohort (Aβ-Stable vs. Aβ-Converter, and to the labels in regression cohort of CSF (Aβ42) and PET(global SUVR).

	ICV	hippocampus	Entorhinal	Fusiform	MidTemp	Ventricles	WholeBrain
**CSF-cohort**	0.04	-0.05	-0.11	-0.15	-0.06	0.25	-0.09
**PET-cohort**	0.02	-0.05	0.06	0.02	0.05	0.1	0.02
**CSF, Regression-cohort**	-0.07	0.16	0.12	0.09	0.06	-0.19	0.01
**PET, Regression-cohort**	0.03	-0.17	-0.1	-0.02	-0.05	0.12	-0.04

Spplementary Figures

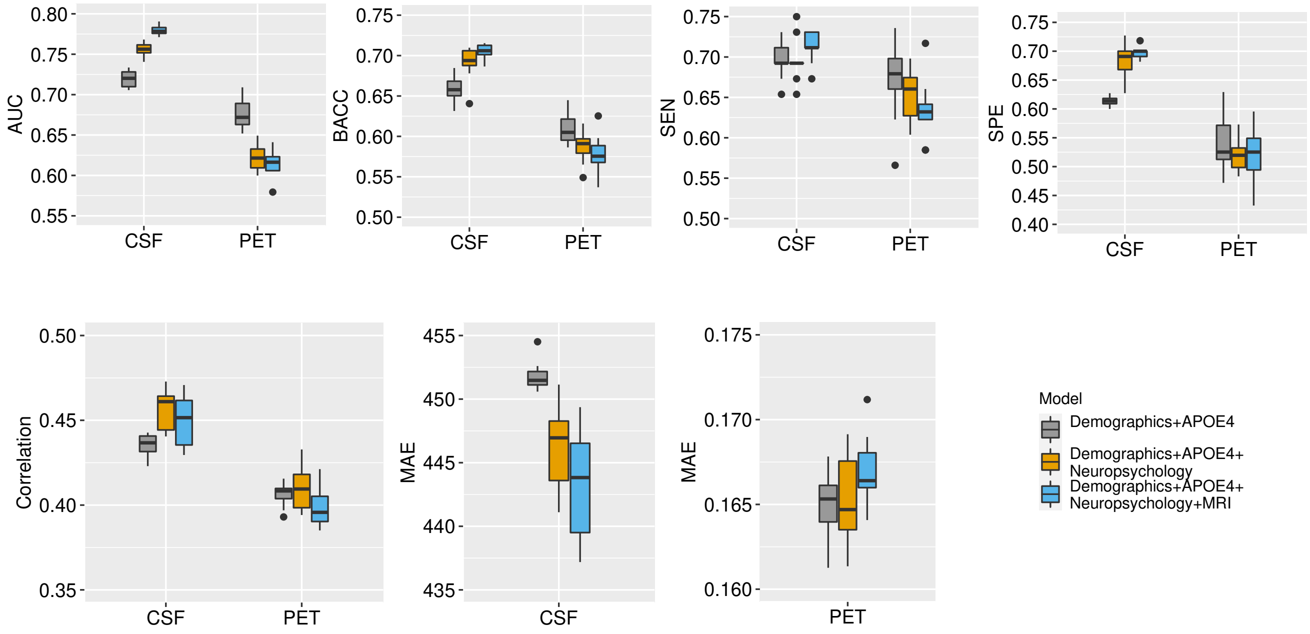

Figure S1: Predicting future Aβ positivity from multimodal data excluding PET and CSF baseline measures: Box plots for AUC, balanced accuracy (BACC), sensitivity (SEN), and specificity (SPE) for predicting Aβ positivity in Aβ negative individuals, and the correlation score and mean absolute error (MAE) for predicting future Aβ42 (CSF) and global SUVR (PET) measures in CSF and PET cohorts. The results are derived from 10 computation runs. In each box, the central mark shown in black is the median, the edges of the box are the 25th and 75th percentiles, whereas the whiskers extend to the most extreme data points not considered outliers, and outliers are plotted as dots.

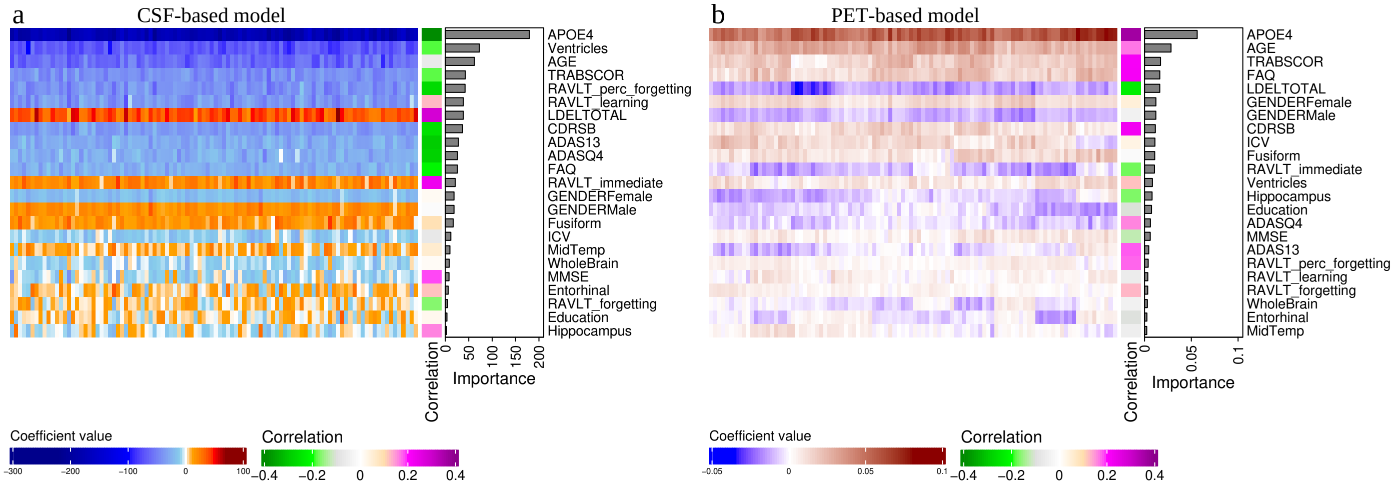

Figure S2: Predicting future Aβ positivity from multimodal data excluding PET and CSF baseline measures: Heatmap of coefficient values across 10 runs of 10-fold CV (100 models) for a) predicting future Aβ42 (CSF-based) and for (b predicting future global SUVR measure (pet-based), with a single column heatmap representing the correlation score between each variable and the label (future Aβ42, future global SUVR), and a bar graph showing the importance of each predictor calculated by the mean of the absolute value of regression coefficients derived by ridge linear regression.

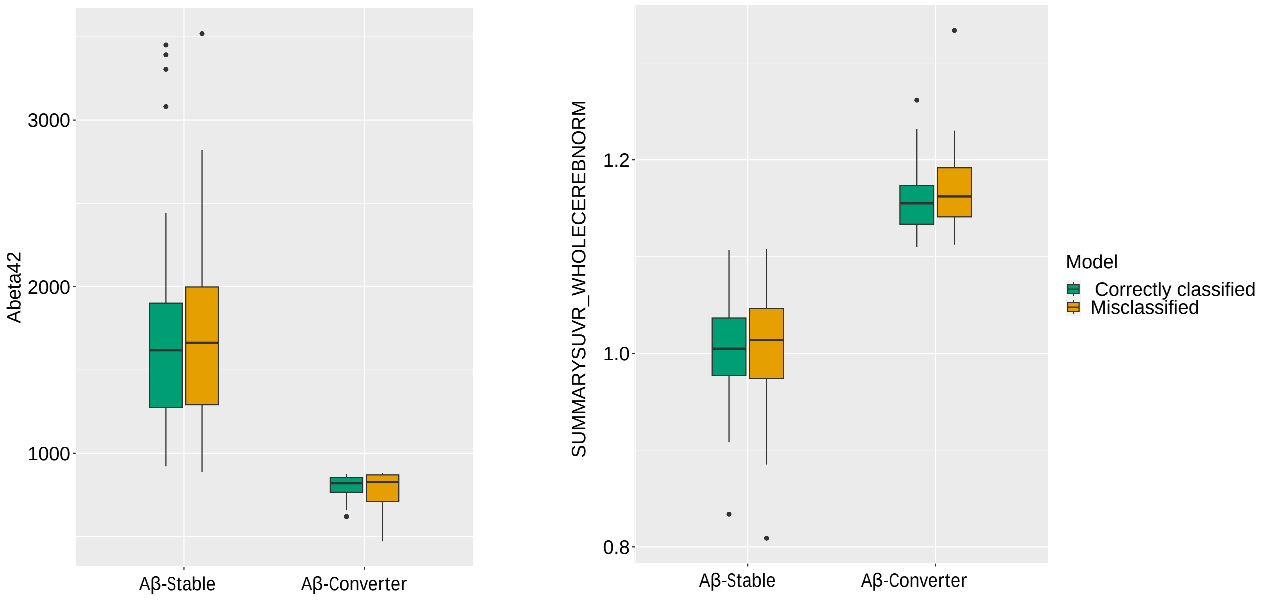

Figure S3: Predicting future Aβ positivity from multimodal data excluding PET and CSF baseline measures: Box plot of Aβ-values from the last visit for Aβ-Stable and Aβ-Converter groups, for correctly classified and misclassified individuals. In the case of Aβ-Converters, the last visit was identified as the one when they converted from negative to positive Aβ. For the Aβ-Stable group, it was simply the last available visit. Given the execution of 10 separate computation runs, each individual has 10 predictions. Individuals misclassified at least 5 times out of 10 runs were considered as misclassified, while those accurately classified at least 6 times out of 10 predictions were considered as correctly classified.

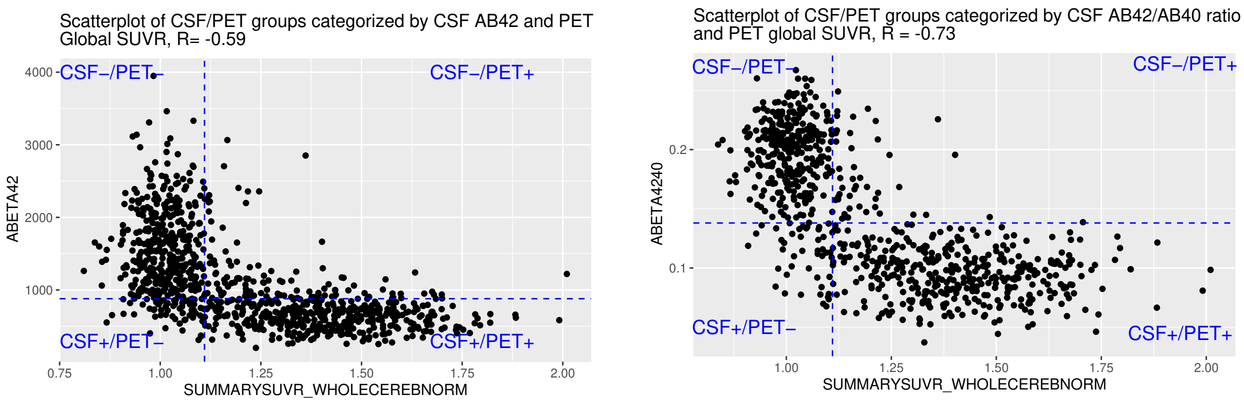

Figure S4: Scatterplots of CSF Aβ42 and the Aβ42/Aβ40 ratio and PET global SUVR.

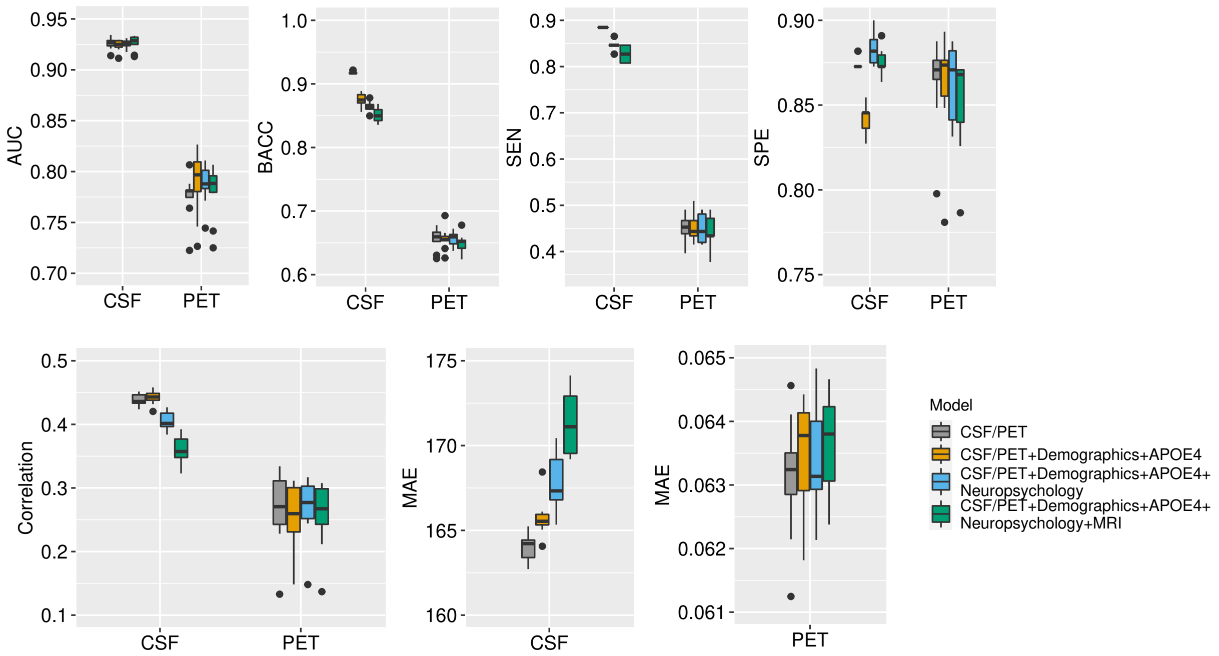

Figure S5: Predicting future Aβ positivity from multimodal data including baseline CSF/PET measures: Box plots for AUC, BACC, SEN, and SPE for predicting Aβ positivity in Aβ negative individuals, and the correlation score and mean absolute error (MAE) for predicting the difference between future and baseline Aβ42 (CSF) and global SUVR (PET) in CSF and PET cohorts. The results are derived from 10 computation runs. In each box, the central mark shown in black is the median, the edges of the box are the 25th and 75th percentiles, whereas the whiskers extend to the most extreme data points not considered outliers, and outliers are plotted as dots. CSF/PET stands for CSF baseline measures (Aβ42, pTau, Tau) for predicting CSF-based Aβ positivity and PET measures (global and regional) for predicting PET-based Aβ positivity

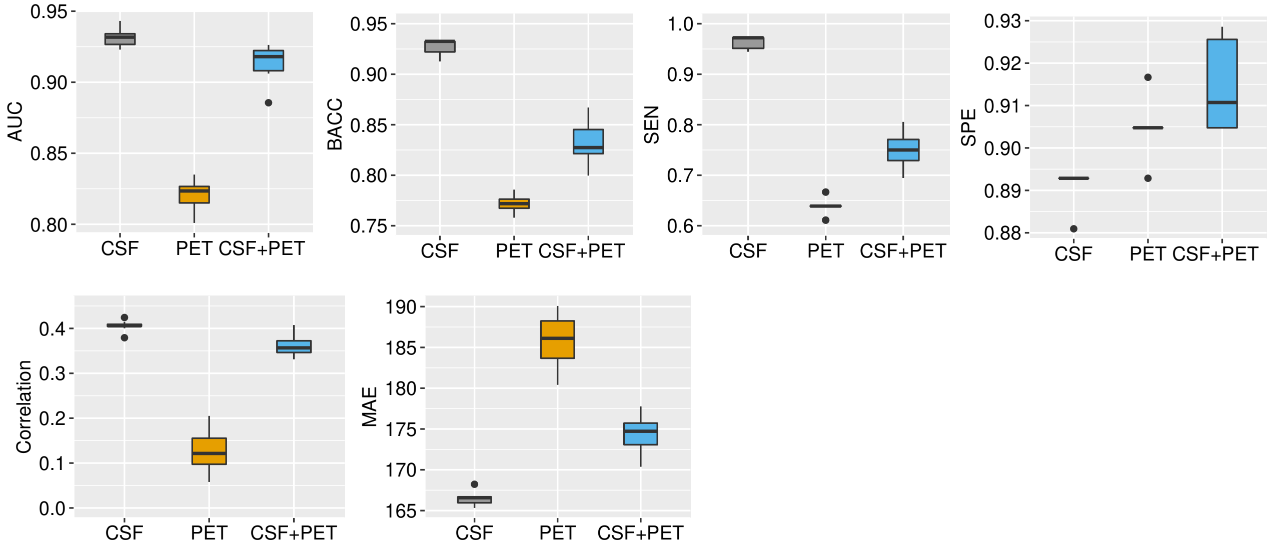

Figure S6: Predicting CSF-based future Aβ positivity from CSF and PET baseline measures: Box plots for AUC, balanced accuracy (BACC), sensitivity (SEN), and specificity (SPE) for predicting Aβ positivity in Aβ negative individuals and the correlation score and mean absolute error (MAE) for predicting the difference between future and baseline Aβ42 (CSF-based). The results are derived from 10 computation runs. In each box, the central mark shown in black is the median, the edges of the box are the 25th and 75th percentiles, whereas the whiskers extend to the most extreme data points not considered outliers, and outliers are plotted as dots. CSF stands for CSF baseline measures (Aβ42, pTau, Tau) and PET stands for PET baseline measures (global and regional).

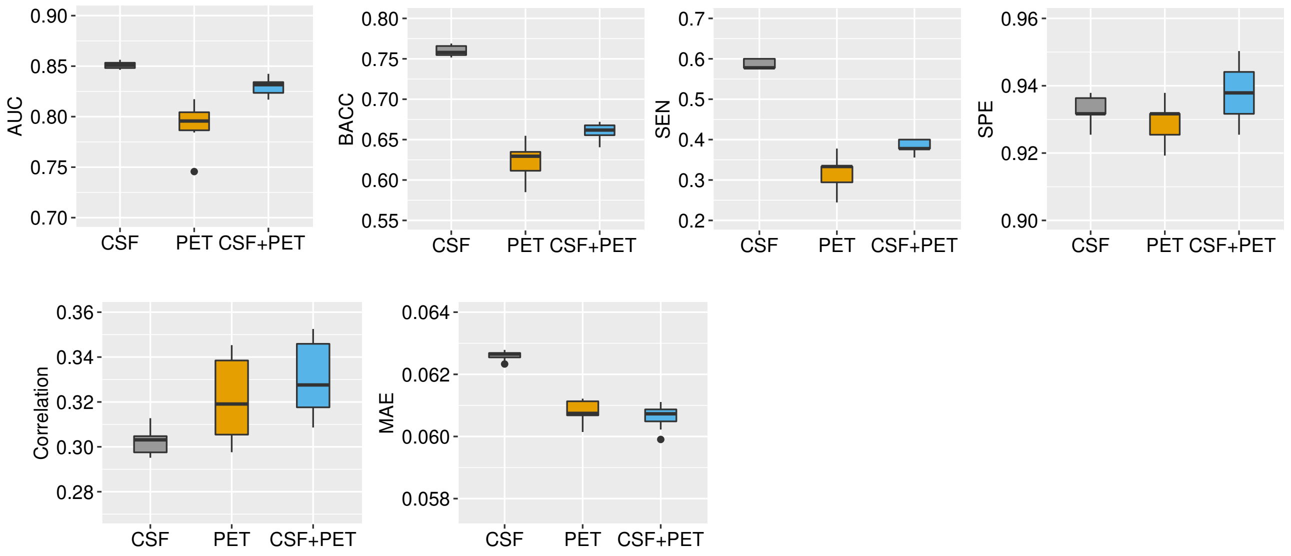

Figure S7: Predicting PET-based future Aβ positivity from CSF and PET baseline measures: Box plots for AUC, balanced accuracy (BACC), sensitivity (SEN), and specificity (SPE) for predicting Aβ positivity in Aβ negative individuals and the correlation score and mean absolute error (MAE) for predicting the difference between future and baseline global SUVR (PET-based). The results are derived from 10 computation runs. In each box, the central mark shown in black is the median, the edges of the box are the 25th and 75th percentiles, whereas the whiskers extend to the most extreme data points not considered outliers, and outliers are plotted as dots. CSF stands for CSF baseline measures (Aβ42, pTau, Tau) and PET stands for PET baseline measures (global and regional).

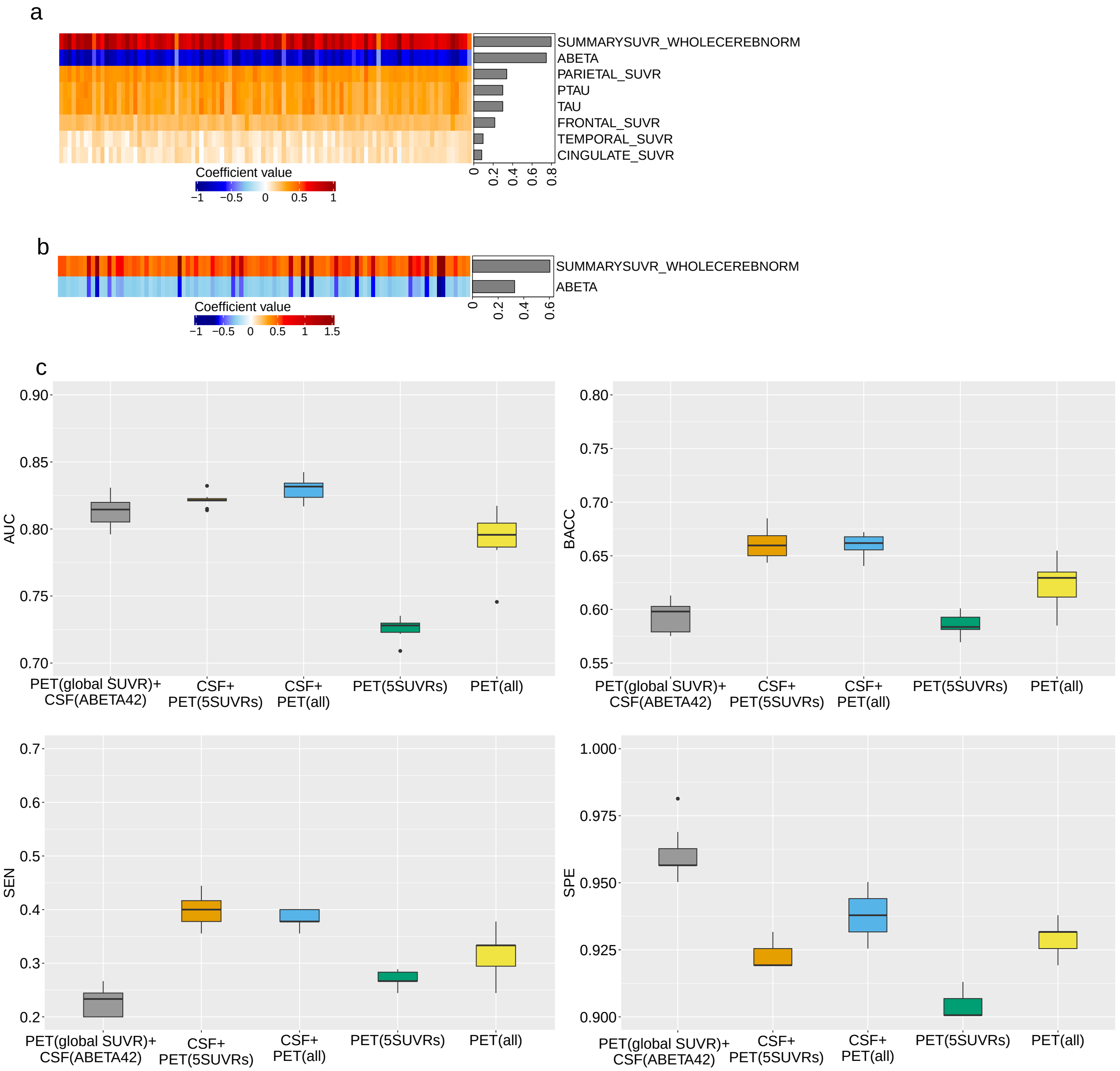

Figure S8: Predicting PET-based future Aβ positivity from CSF and PET baseline measures: a,b) Heatmap of coefficient values across 10 runs of 10-fold CV (100 models) for PET-based classification model using a) baseline CSF measures and 5 SUVRs and b) baseline CSFAβ42 and global SUVR, c) Box plots for AUC, balanced accuracy (BACC), sensitivity (SEN), and specificity (SPE) for predicting Aβ-positivity for comparison between using different number of SUVRs for prediction PET-based Aβ-positivity. The results are derived from 10 computation runs.
